# Concomitant aortic valve repair for aortic insufficiency and implantation of left ventricle mechanical support

**DOI:** 10.1111/jocs.16547

**Published:** 2022-04-26

**Authors:** Arun K. Singhal, Jarrod Bang, Anthony L. Panos, Andrew Feider, Satoshi Hanada, J. Scott Rankin

**Affiliations:** ^1^ Department of Cardiothoracic Surgery University of Iowa Hospitals & Clinics Iowa City Iowa USA; ^2^ Department of Anesthesia University of Iowa Carver College of Medicine Iowa City Iowa USA; ^3^ Department of Cardiovascular and Thoracic Surgery WVU Heart and Vascular Institute, West Virginia University Morgantown West Virginia USA

**Keywords:** aortic valve regurgitation, aortic valve repair, complications, HAART Ring, heart failure, LVAD, nonischemic cardiomyopathy

## Abstract

**Background:**

Moderate to severe aortic valve insufficiency (AI) in patients undergoing left ventricular assist device (LVAD) implantation is a significant complication which occurs in up to 10.7% of patients in the INTERMACS database and has profound consequences for survival. Preoperative Impella use is associaed with greater post‐LVAD AI.

**Case Presentation:**

56 y/o Caucasian female with acute exacerbation of chronic congestive heart failure who needed urgent Impella placement followed by elective Heartmate III LVAD.

**Conclusion:**

Patients who have aortic valve regurgitation at the time of implantation have been handled by several methods, including aortic valve leaflets approximation, to aortic valve replacement or even valve closure. We report a case of geometric ring annuloplasty for repair of a regurgitant aortic valve during destination LVAD implantation.

**Abbreviations**
AI: aortic value insufficiency
AV: aortic valve
DT: destination therapy
EF: ejection fraction
LV: left ventricle
LVAD: left ventricular assist device
TEE: transesophageal echo

## 1 INTRODUCTION

Aortic value insufficiency (AI) is a significant complication of left ventricular assist devices (LVAD). In a recent INTERMACS registry analysis, 10.7% of patients developed moderate to severe AI in a time dependent fashion and this was associated with worse hemodynamics, increased hospitalization and decreased survival.^1^ Since AI is unfavorable in this population, mitigation strategies have been developed. The first is prediction of patients who may develop AI which include mild AI pre‐LVAD, smaller body surface area, older age, female gender, and dilated aortic roots.^2^ Additionally, Impella placement may be a separate risk factor for AI in durable LVADs. In a single institution study of 41 patients requiring Impella immediately before durable LVAD (HeartMate 2 and 3), 82% of recipients developed mild to moderate AI compared to 43% in those without Impella.^3^ Surgical treatment strategies fall include aortic valve replacement (AVR), central oversewing (Park's stitch) or valve closure in selected cases.^4^, ^5^, ^6^ However, replacement with bioprosthetic AVR has risk of valve thrombosis. Central oversewing is effective but risks sudden death in case of LVAD stoppage and may complicate assessment of left ventricular function. Furthermore, a permanently closed or nonopening valve has increased prevalence of AI compared to either intermittent or complete opening of the aortic valve (AV).^7^ No strategy is demonstrably optimal.

**Figure 1:**
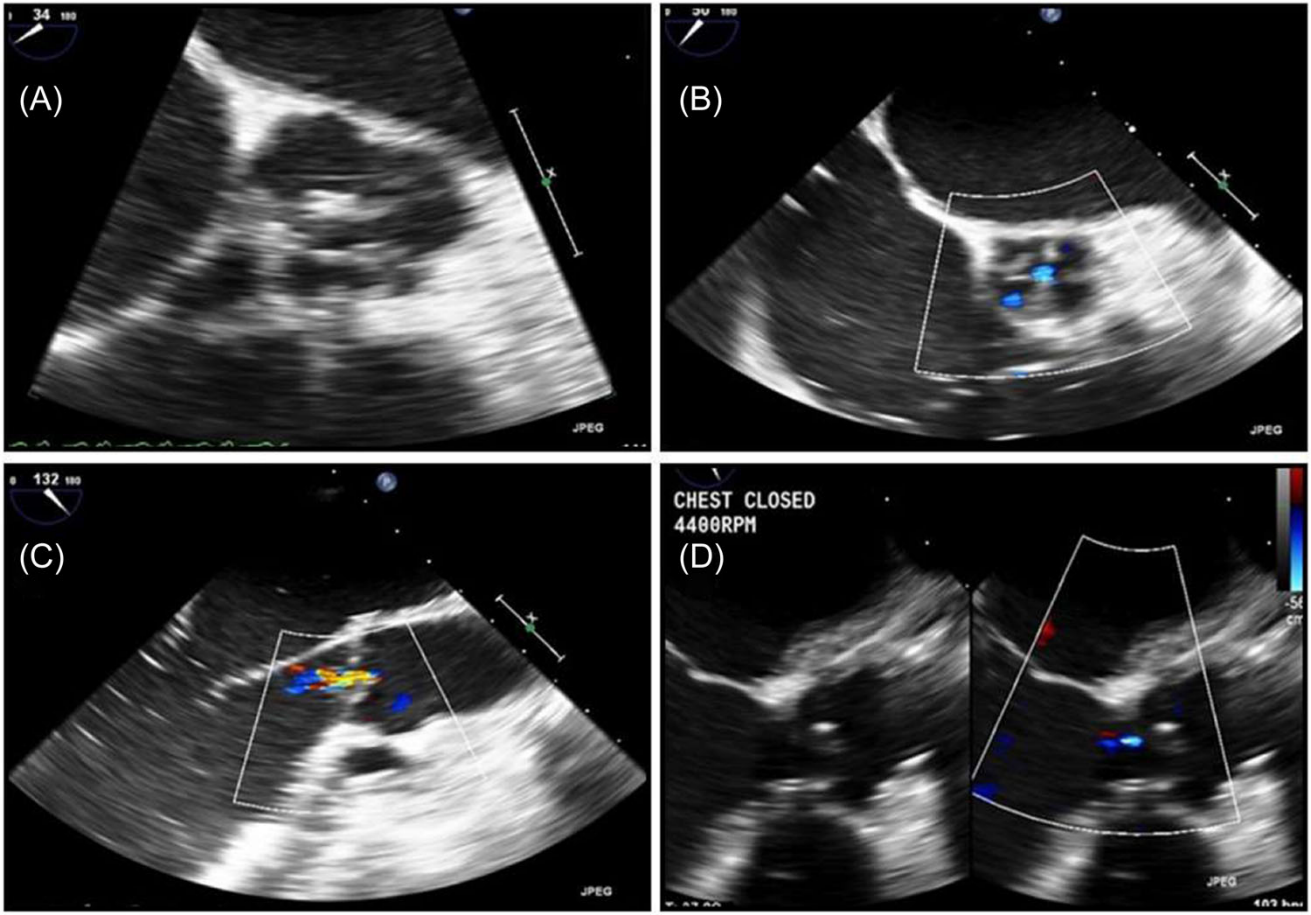
(A) Precardiopulmonary bypass, mid‐esophageal aortic valve short axis view with Impella in place crossing the aortic valve between the noncoronary and right coronary cusps. (B) On cardiopulmonary bypass following removal of Impella device, mid‐esophageal aortic valve short axis with color flow doppler demonstrating two AI Jets, one central and one at the location of the removed Impella. (C) On cardiopulmonary bypass following removal of Impella catheter, mid‐esophageal aortic valve long axis view demonstrating the central aortic insufficiency jet. (D) Postcardiopulmonary bypass and aortic valve repair, mid‐esophageal aortic valve long axis view demonstrating trace aortic insufficiency through a central jet

AV repair and associated leaflet reconstruction is reported during LVAD implantation in a 56‐year‐old Caucasian female with acute exacerbation of her chronic congestive heart failure. Her history is significant for biventricular reduced ejection fraction (EF) secondary to prior chemotherapy, single vessel left anterior descending artery stenosis with previous stent, left bundle branch block, Type 2 diabetes mellitus and hyperlipidemia. Her LV EF was 15%. She was admitted, started on inotropes, and support was escalated to an Impella CP. She developed high panel reactive antigens and was offered a Heartmate 3 LVAD as Destination therapy. Preoperative transesophageal echo (TEE) revealed an Impella CP device across the AV between the noncoronary and right coronary cusps. Initially no aortic insufficiency (AI) was visualized. However, after initiation of cardiopulmonary bypass and removal of the Impella, AI of mild severity was identified (Figure 1). Two separate AI jets were present, a larger central jet and a smaller commissural jet in the location previously occupied by the Impella device.

## 2 METHODS

**Figure 2:**
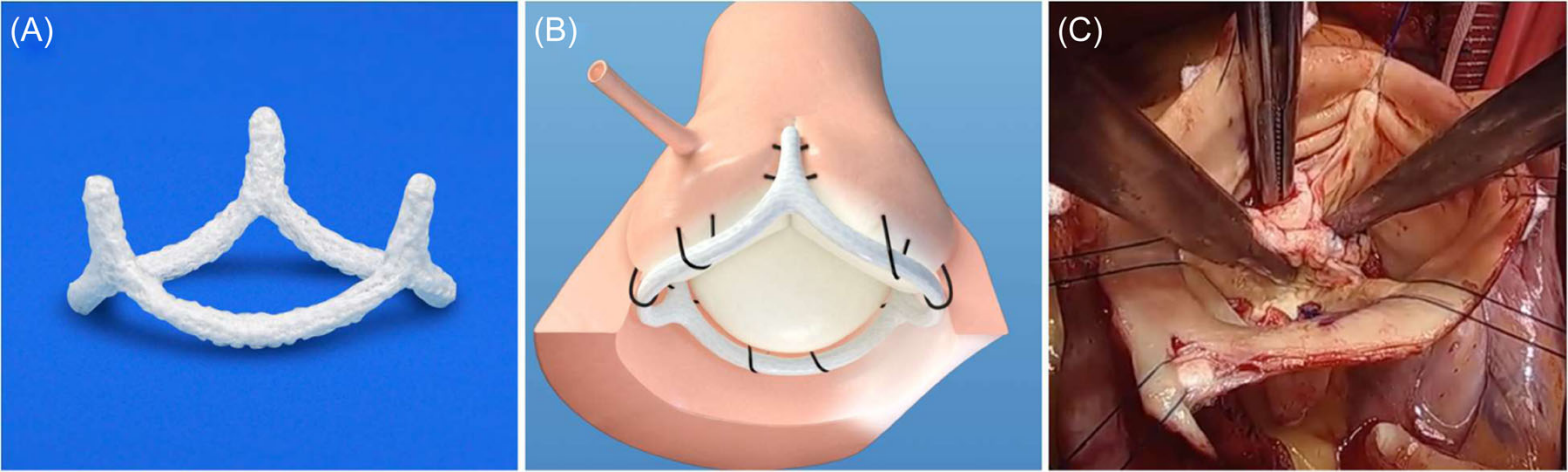
(A) Aortic annuloplasty ring (HAART 300—trileaflet aortic valve). (B) Illustration of aortic annuloplasty ring in the sub valvular position. (C) Aortic valve postrepair with aortic annuloplasty ring

Impella CP was placed via right axillary artery cutdown with fluoroscopic and TEE guidance (Abiomed).^8^ The patient was scheduled for HeartMate 3 LVAD (Abbott Il) with inflow from the left ventricle apex and outflow on the right lateral wall of the Aorta.^9^ Before the outflow anastomosis, the aortic value was repaired in the operating room using a 19 mm aortic annuloplasty ring (HAART 300, BioStable Science and Engineering) (Figure 2). The ring was sutured under the valve annulus using trans‐annular horizontal mattress sutures of 3‐0 Tycron (Teleflex Medical). Cabrol‐like mattress sutures buried the ring posts into the sub‐commissural triangles. The ring was passed below the valve, and looping sutures secured each sinus aspect to the corresponding annulus. The ring posts were positioned low in the subcommissural triangle to raise the commissural tops relative to the base, and increase leaflet vertical coaptation height.^10^ Noncoronary‐cusp prolapse was corrected by leaflet plication. After completion of the outflow to the right lateral wall of the Aorta, a surgical centrimag RVAD was placed. We clamped the aorta, delivered cardioplegia, performed the AV repair and the LVAD graft anastomosis to the ascending aorta during one cross‐clamp period. Cardiopulmonary bypass time was 196 min and aortic cross‐clamp time was 96 min. After AV repair and LVAD implantation, TEE revealed a well seated annuloplasty ring and trivial AI with a small central jet (Figure 1). The AV leaflets did not open during systole postoperatively so a postoperative gradient could not be obtained. However, at 3‐month follow‐up the AV is opening without evidence of AI. Unfortunately, echo windows were inadequate to calculate AV gradient.

## 3 DISCUSSION

Our patient has multiple risk factors for development of AI in the first year: low BSA, female gender and Impella support before LVAD. Based on various reports, her estimated risk for developing moderate to severe AI in the first year is between 14% and 82%. Options for AI prevention include AVR, Park's stitch and oversewing the valve.^4^, ^5^, ^6^ Our choices also include no intervention. Because of our comfort with AV repair, we chose the alternative of a ring based repair. One hypothesis for post durable LVAD AI is the large transvalvular gradient (high in the Aorta and low in the LV) resulting in increased shear stress on the AV and annular dilation. As presented by Carpentier, the role of an internal geometric annuloplasty ring is to reduce or stabilize annular dimension over the long term. We believe that the specific role of our intervention in this case is stabilization of the annulus to prevent long term dilation due to from the long term retrograde stress from the LVAD. Furthermore, early data suggest that valve‐related complications are low, and ring annuloplasty is a simple and useful component of AV repair.^10^, ^11^ Last, the AV is not restricted from opening, if ventricular function is adequate.^7^

In summary, we report the first use of the HAART AV repair to address AI during LVAD placement. In doing so we were able to perform a durable repair of the valve without either surgical closure of the AV or AVR. We plan to follow this patient serially and consider repair in future patients with AI requiring LVAD.

## AUTHOR CONTRIBUTIONS

**Arun K. Singhal, Jarrod Bang, Anthony L. Panos, Andrew Feider, Satoshi Hanada, and J. Scott Rankin**: were involved in the design and analysis of the case, the original draft, and revised drafts of the paper and gave final approval of the version to be published.

## CONFLICTS OF INTEREST

Dr. Rankin is a consultant for BioStable Science and Engineering, Austin TX.
